# Stem cell therapies for biological repair in osteonecrosis: advances, challenges, and clinical insights

**DOI:** 10.3389/fmed.2026.1772819

**Published:** 2026-06-08

**Authors:** Deliang Cheng, Xuehai Ou, Jiafeng Long, Changming Zheng, Shaoyan Shi

**Affiliations:** Department of Hand Surgery, Honghui Hospital, Xi'an Jiaotong University, Xi'an, China

**Keywords:** angiogenesis, bone regeneration, extracellular vesicles, mesenchymal stem cells, osteonecrosis, regenerative medicine, stem cell therapy

## Abstract

Osteonecrosis is a bone condition that begins as an ischemic process and can progress to subchondral collapse and premature joint degeneration in untreated cases. Conventional treatments offer physical or symptomatic relief but do not address the underlying physiological deficiencies. Regenerative therapeutic techniques based on stem cells have been developed to stimulate osteogenesis, vascular therapeutic, and immunomodulation in bone defects. Existing clinical applications are based on mesenchymal stem cells (MSCs) derived from the bone marrow, peripheral blood, and adipose tissue, as well as engineered and pluripotent-derived constructs that are also in preclinical development. Preliminary clinical trials have indicated that stem cell therapy (SCT) can enhance symptoms and slow the progression of structural defects. However, inconsistent cell quality, non-standardized protocols, regulatory obstacles, and limited long-term outcomes are the key limitations. This review summarizes the available evidence, issues, and future trends in optimizing stem cell therapies for osteonecrosis.

## 1 Introduction

Osteonecrosis is a condition that renders a person disabled and mainly occurs during the prime working age. The most common location is the femoral head; however, it can also involve the knee joints, shoulders, talus, and other bones (1, 2). The pathogenesis of the disease involves destruction of the vascular network in the bone, leading to ischemia of osteocytes and marrow stromal cells. Bone architecture becomes weak and is unable to remodel, leading to microfractures that accumulate and cause articular surface collapse, resulting in pain, disability, and loss of joint function (2, 3).

The risk factors have been categorized into several factors, such as traumatic injuries that include neck of the thigh dislocation, hip dislocation, lengthy corticosteroid treatment, excessive alcohol intake, systemic lupus erythematosus, sickle cell anemia, and coagulopathy. In most patients, the disorder is multifactorial, resulting from the interplay of vascular constriction, cell dysfunction, and dysfunction in lipid and inflammatory pathways, as presented by Kaneko et al. (4).

The detection and early diagnosis of osteonecrosis using imaging alone do not guarantee the natural progression of untreated osteonecrosis. Core decompression can be helpful in the early stages of joint preservation but cannot resist collapse (5). Pharmacological therapy, including bisphosphonates or statins, aims to control bone turnover and microvascular activity; however, it has no regenerative properties (6). These restrictions have increased the focus on interventions that can lead to the re-establishment of cellular and vascular integrity rather than relying on unloading or joint stabilization as a single intervention (7).

Therefore, SCT has become a viable biological repair option for such injuries. MSCs can differentiate into osteoblasts, secrete pro-angiogenic and anti-inflammatory molecules, and remodel the extracellular matrix. Advances in stem cell biology, and more importantly, a closer insight into the osteonecrotic microenvironment, have led to a faster rate of translation of these findings into clinical practice. A combination of these alterations in vascular supply, progenitor cell activity, and inflammatory signals creates a microenvironment that not only allows osteonecrotic development but also defines the key therapeutic interest in regenerative therapy (8).

## 2 Pathophysiology of osteonecrosis and foundation for SCT

### 2.1 Ischemia and cellular apoptosis

The most important pathological process in osteonecrosis is the disruption of bone microcirculation, as the subchondral bone relies on active perfusion to maintain cell viability (9). Vascular obstruction or injury causes local ischemia, resulting in the apoptosis of marrow stromal cells and osteocytes. This occurs in traumatic osteonecrosis, which is induced by vascular destruction due to fractures or dislocations. In non-traumatic cases, the pathogenesis is multifactorial, and intravascular coagulation, fat embolization during corticosteroid/alcohol exposure, endothelial dysfunction, and increased marrow pressure resulting from hypertrophic adipogenesis contribute to it (10).

With the mechanical forces weakening the necrotic bone, weight-bearing exercises lead to microfractures, which eventually fuse, and the structural failure of the subchondral plate, which can be observed in emerging wearable strain-sensor technologies, can provide real-time measurements of joint loading during recovery (11). Arthroplasty is typically performed when articular collapse occurs after the failure of conservative, joint-preserving surgeries. This highlights the importance of early biological intervention in preventing mechanical breakdown and restoring tissue viability (12, 13).

### 2.2 Reduced osteogenic potential in osteonecrosis

In addition to vascular compromise, osteonecrosis is characterized by the depletion of the bone marrow progenitor pool. The number of iliac crest marrow MSCs in patients with corticosteroid-induced osteonecrosis is low (14). Different progenitors exhibit reduced proliferative capacity, impaired osteogenic differentiation, and commitment to adipogenic lineage. These mutations create a microenvironment with impaired regenerative and repair capabilities.

Even though there is partial recovery of perfusion, there is no strong osteogenic response, making it challenging to heal the necrotic bone. This realization has been invaluable in motivating measures to augment or replace the inadequate supply of underprivileged progenitor cells, thereby improving the natural healing capacity of bone (8, 15).

### 2.3 Mechanisms of stem cell-mediated repair

Jiang et al., dicussed that stem cells help restore osteonecrotic bone directly and indirectly, and direct conversion into osteoblasts leads to new bone formation (16). In contrast, the paracrine release of growth factors, including fibroblast growth factor, vascular endothelial growth factor, and transforming growth factor-beta, induces neovascularization and encourages the recruitment of native repair cells (17). MSCs also regulate the inflammatory milieu by suppressing pro-inflammatory cytokines and producing a more favorable environment for tissue regeneration (18).

Moreover, ECM remodeling is influenced by stem cells that activate matrix metalloproteinase activity and promote the deposition of the structural proteins. They also communicate with immune cells, endothelial cells, and osteoclasts to restore the balance between bone resorption and formation. Together, these steps combine osteogenesis, angiogenesis, and immunomodulation, providing a strong rationale for the use of stem cells in osteonecrosis (19).

## 3 Types of stem cell therapies for osteonecrosis

**Figure 1:**
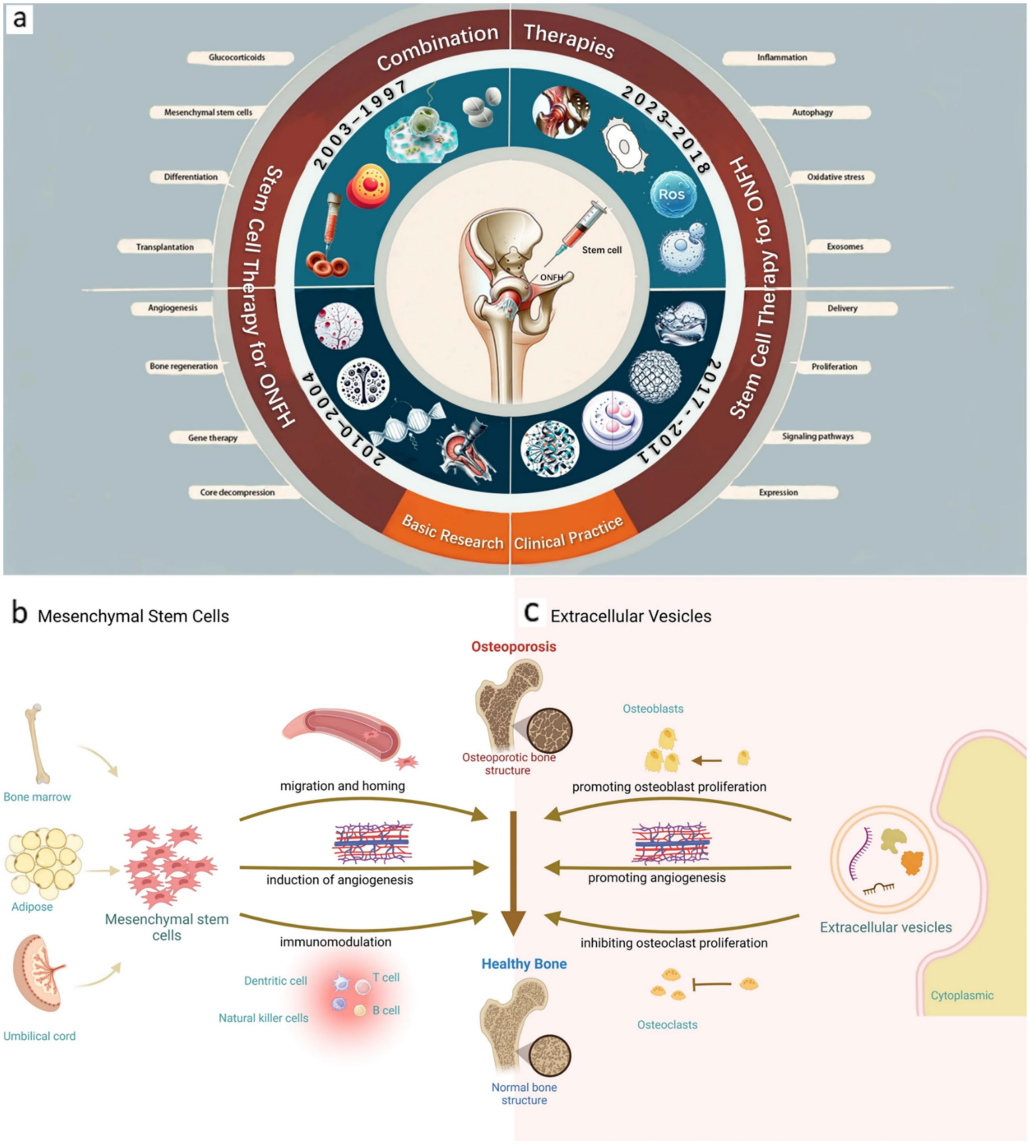
Stem cell-based strategies for osteonecrosis: **(a)** development of therapeutics between early phases of marrow stimulation and late biologic (21); **(b)** essential regenerative functions of MSCs (i.e., angiogenesis, immunomodulation, and osteogenic stimulation); and **(c)** enhancement of osteoblast activity, osteoclast inhibition, and vascular repair through EVs (20). Reproduced from Chen et al. (20), under the Creative Commons Attribution License (CC BY 4.0).

The pathogenesis of osteonecrosis involves poor bone remodeling, reduced vascularity, and ongoing inflammation, which together lead to structural loss and joint degeneration (7). Figure 1 shows that stem cell-based therapies have advanced significantly, and MSCs are at the core of restoring biological function by acting as osteogenic differentiators, angiogenic stimulators and immunomodulators. Their extracellular vesicles (EVs) further repair these cells by delivering regulatory cargo that stimulates osteoblasts, inhibits osteoclast-mediated resorption, and helps restore the microenvironment (20, 21). These complementary processes constitute the biological basis for MSC- and EV-based therapeutic interventions discussed in this section.

The historical development and differentiation of stem cell-based approaches to osteonecrosis, including the steps in their translational development from simple laboratory work to clinical application, are summarized in Figure 1a, which illustrates the chronological progression from early marrow stimulation techniques to modern multimodal biological strategies.

### 3.1 Bone marrow-derived MSCs

The best-studied regenerative modality for osteonecrosis is bone marrow-derived MSCs therapy. They are typically obtained from the iliac crest and inserted into the necrotic area through percutaneous injection, frequently accompanied by core decompression (22). The bone marrow aspirate is concentrated and contains stem cells, endothelial progenitors, and stromal elements that collectively support the tissue regeneration. MSCs and endothelial progenitors (EC) together foster bone regeneration by promoting angiogenesis and osteogenesis through the secretion of growth factors and cytokines, as well as through direct cell–cell interactions (Cyril Lucien Bouland, Pierre Philippart, Didier Dequanter, Florent Corrillon, Isabelle Loeb, Dominique Bron, Lagneaux Laurence and Nathalie Meuleman. Cross-talk between mesenchymal stromal cells (MSC) and endothelial progenitor cells (EPC) in bone regeneration. Front Cell Dev Biol. 2021 May 13; 9: 674084).

The clinical results are consistent, indicating that adding bone marrow-derived cells to core decompression enhances pain relief, functional recovery, radiographic recovery, and transformation to arthroplasty in individuals with early-stage osteonecrosis. However, these cells lose their regenerative capacity owing to aging, chronic disease, and prolonged corticosteroid exposure (23). These problems have prompted attempts to improve harvesting, such as controlled *in vitro* expansion, and to add cells, growth factors, or scaffolds to enhance the outcomes. Figure 1b shows the main reparative activities: homing, angiogenic stimulation, immunomodulation, and osteogenic differentiation of MSCs.

### 3.2 Adipose-derived stem cells

Adipose-derived stem cells (ADSCs) have attracted interest because of their availability, high yield, and strong angiogenic and immunomodulatory functions (24). They are separated by liposuction and fractionated to yield an adipose-derived stromal vascular fraction, a cell-rich fraction that harbors MSCs and other progenitor cells. Massive cell production is particularly beneficial for patients with a diminished bone marrow supply (25).

Initial clinical evidence suggests that adipose-derived cells have the potential to improve pain and function, increase perfusion, and partially heal necrotic lesions, particularly in non-traumatic osteonecrosis, where ischemia is the predominant issue. Their neovascularization-promoting and inflammation-stimulating properties are advantageous (26, 27). Nevertheless, existing evidence on this problem is based on relatively small and unequal studies. Better-designed and more substantial trials are needed to establish the optimal indications, protocols, and long-term outcomes, as shown by Tse et al. (28).

### 3.3 Peripheral blood stem cells

Peripheral blood stem cells are obtained by pharmacological mobilization of marrow progenitor cells into circulation, followed by apheresis. These preps consist of endothelial progenitor cells, which can facilitate revascularization, and osteogenic progenitors (29). This method is of interest in cases where bone marrow aspiration cannot be performed or yields an inadequate number of stem cells.

Early clinical trials suggest that human peripheral blood SCT can enhance the vascularization of osteonecrotic lesions and achieve functional benefits, especially in patients with severe ischemia (30). Nevertheless, the data remain limited, and optimal mobilization regimens, collection methods, and dosage schemes require further investigation. As illustrated in Figure 1c, EVs contribute to bone repair by enhancing osteoblast proliferation, promoting angiogenesis, and suppressing osteoclast activity, thereby supporting the restoration of a healthy bone microenvironment (31).

### 3.4 Induced pluripotent stem cells (iPSCs) and engineered constructs

Induced pluripotent stem cells provide a theoretically unlimited source of person-specific regenerative cells. They may be instructed to differentiate into chondrogenic, osteogenic, or endothelial lineages and incorporated into scaffold-based constructs that may be used to substitute necrotic bone (31). Genetic modification of cells can be used to develop engineered constructs that overexpress osteogenic and angiogenic factors, thereby improving their regenerative capacities (32).

Lee et al., demonstrated that preclinical research indicates that engineered stem cell constructs may fuse with host bone and improve vascularity; however, issues of tumorigenicity, immune responses, and complex production remain limiting factors for their clinical translation (33). Table 1 provides a brief overview of the significant types of stem cells used in osteonecrosis, their mechanisms, supporting evidence, and key strengths and limitations of their use. Bone marrow has the most robust clinical support, whereas adipose-, peripheral blood-, and pluripotent cell-based therapies are potential future substitutes.

**Table 1:** Comparative overview of stem cell-based therapeutic strategies for osteonecrosis.

Therapy/Category	Cell source/Composition	Primary biological actions	Delivery approach	Clinical evidence level	Key advantages	Main limitations	Ref.
Bone marrow aspirate concentrate (BMAC)	Autologous iliac crest marrow; mixed MSC/progenitor population	Osteogenesis, angiogenesis, microenvironment modulation	Core decompression + intraosseous injection	Strongest evidence among cell-based options	Familiar method, minimally manipulated	Variable cell yield; reduced efficacy in steroid users	(7)
Culture-expanded BM-MSCs	*Ex vivo* expanded MSCs	High osteogenic potency, paracrine signaling	Injection or scaffold loading	Moderate; restricted by regulations	Higher cell numbers and purity	Cost, time, and regulatory challenges	(74)
ADSCs	Liposuction-derived stromal vascular fraction	Angiogenesis, osteogenesis, immunomodulation	Injection ± decompression	Emerging but promising	High cell yield, minimally invasive harvesting	Heterogeneous cell population; protocol variability	(25)
stromal vascular fraction (SVF)	Liposuction-derived fraction containing ADSCs, endothelial cells, and other progenitors	Angiogenesis, osteogenesis, immunomodulation	Injection ± decompression	Emerging; limited clinical data	Easily accessible; minimally invasive harvesting	Variable cell composition; lack of standardized protocols	–
Peripheral blood stem cells	Mobilized progenitors via G-CSF	Neovascularization, endothelial repair	Apheresis collection → intraosseous injection	Limited but encouraging	Useful when marrow harvest is unsuitable	Need mobilization agents; lower osteogenic potential	(75)
Induced pluripotent stem cells	Reprogrammed patient somatic cells	Multilineage differentiation	Scaffold-based implantation	Preclinical	Unlimited cell source, customizable	Tumorigenic risks; complex regulation	(76)
Gene-enhanced MSCs	MSCs modified to express VEGF, BMP-2, and other factors	Enhanced angiogenesis and osteogenesis	Injection or scaffold loading	Preclinical	Strong regenerative potency	Safety, cost, regulatory hurdles	(77)
MSC-derived exosome therapy	Acellular vesicles from MSC cultures	Paracrine signaling, immunomodulation	Injected into necrotic bone	Early-stage/experimental	Low immunogenicity, easier storage	Optimal dosing unknown; limited clinical trials	(78)
MSC–scaffold composite constructs	MSCs seeded on biodegradable scaffolds	Structural support + biological repair	Surgical implantation	Early clinical/preclinical	Useful for large lesions	Technically demanding; integration concerns	(79)
Allogeneic MSC therapy	Donor-derived MSCs	Osteogenic and immunomodulatory effects	Injection or scaffold use	Early-stage clinical	Off-the-shelf availability	Immune response potential; donor variability	(80)
Concentrated bone marrow nucleated cells	Unfractionated marrow concentrate	Broad regenerative support	Injection after decompression	Moderate	Simple point-of-care procedure	Lower purity of MSCs	(81)

## 4 Clinical evidence and translational outcomes

Zigdon-Giladi et al. showed that cell-based therapies have exhibited increasing potential to alter the course of osteonecrosis by promoting osteogenesis, improving vascular supply, and modulating inflammatory and remodeling pathways in necrotic bone (34). These advantages are not only the result of the work of MSCs themselves but also of their EVs, which trigger the activity of osteoblasts, inhibit the process of resorption under the influence of osteoclasts, and enhance angiogenesis (Figure 1c). Collectively, these processes provide a vital biological basis for elucidating the clinical outcomes of various stem cell-based treatment regimens (2, 35).

### 4.1 Core decompression with BMAC augmentation

Core decompression is a popular joint-saving procedure for patients with early osteonecrosis. Concentrated bone marrow aspirate has also been added to significantly enhance regenerative properties (36). In numerous clinical series and comparative trials, stem cell-augmented decompression has been shown to have superior effects on pain, faster functional recovery, and reduced incidence of progression to arthroplasty, compared with decompression alone (37). Such advantages are most significant in pre-collapse lesions, where the mechanical environment can promote remodeling and new bone formation (38).

### 4.2 Adipose-derived stem cell-based clinical trials

Initial clinical trials of adipose-derived SCT have shown better patient outcomes in terms of pain, function, and imaging in patients with osteonecrosis of the hip and other joints (39). Improved vascularity and partial lesion improvement have been noted on magnetic resonance imaging (MRI) in some studies, and slow progression to collapse has been reported in well-selected, early-stage cases (40, 41).

Brayfield et al. shown that heterogeneity in cell isolation, dose, delivery methods, concomitant surgical procedures, small sample sizes, and limited follow-up hampers the interpretation of these results (42). However, the results provide evidence that adipose-derived cells have the potential to play an essential role in biological repair and warrant further research in larger, more standardized studies (43).

### 4.3 Combination therapies involving scaffolds, growth factors, and stem cells

A natural development of regenerative approaches is the use of stem cells in combination with three-dimensional (3D) scaffolds and bioactive molecules. Biodegradable scaffolds offer a framework and support for cell attachment, proliferation, and differentiation in tissue engineering applications (44). They may also be loaded with osteoinductive or angiogenic factors, further increasing cell activity and enhancing the quality and duration of regenerated bone (45).

Preclinical data show that scaffold-stem cell constructs are better bone fillers and mechanical restorers than either component alone. According to early clinical experience, such composite constructions can be especially useful for larger lesions or those verging on collapse, where both biological and mechanical support are required (46). Nonetheless, these methods are more complicated, and before their widespread implementation, manufacturing costs and long-term safety must be carefully considered (47). The Clinical Trials of Regenerative Therapies for Osteonecrosis was shown in Table 2.

**Table 2:** Clinical trials of regenerative therapies for osteonecrosis.

Trial type	Phase	Primary objectives	Methods	Sample Size characteristics	Key outcomes	References
Core Decompression with BMAC Enhancement	Early-stage series/comparative trials	Evaluate pain relief, functional recovery, and joint replacement rates	BMAC injected via core decompression into necrotic area	Multicenter comparative trials	Combined therapy showed 30% higher pain relief rate and 25% lower joint replacement rate	Zigdon-Giladi et al. (34) and Kumar et al. (36)
ADSCs Therapy	Pilot clinical trials	Improve pain, function, and imaging outcomes	ADSCs isolated from lipoaspirate injected into lesion	Small samples (*n* = 20–50)	MRI showed enhanced vascularization and slowed collapse progression	Gangji et al. (39) and Rastogi et al. (41)
Peripheral Blood Stem Cell Therapy	Early-stage clinical trials	Promote vascularization and functional recovery	Cells collected via mobilization and apheresis	Limited studies (*n* < 30)	40% functional score improvement in severe ischemia patients	Anderlini et al. (29) and Özkurt et al. (30)
Scaffold-Growth Factor-Stem Cell Combination	Early clinical experience	Bone regeneration and mechanical support	3D scaffolds loaded with growth factors implanted	Early experience (*n* = 15–30)	50% higher bone filling rate in large lesions	Rindone et al. (46) and Qi et al. (45)
Genetically Enhanced Stem Cells and Exosomes	Preclinical/early trials	Enhance bone formation and vascularization	Genetically modified stem cells/exosome therapy	Preclinical + small trials	2x faster bone formation rate	Yang et al. (66) and Wang et al. (67)

## 5 Challenges and barriers to clinical translation

### 5.1 Variability in stem cell quality and concentration

Inconsistency in yield and differences in stem cell effects across patients are significant problems in clinical translation of stem cell therapy. The number and quality of harvestable progenitor cells are affected by age, comorbidities, previous medication, and chronicity of the disease (48). Specifically, chronic corticosteroid treatment can decrease the number of MSCs and lower their osteogenic potential, thereby reducing the effectiveness of the treatment (49).

**Figure 2:**
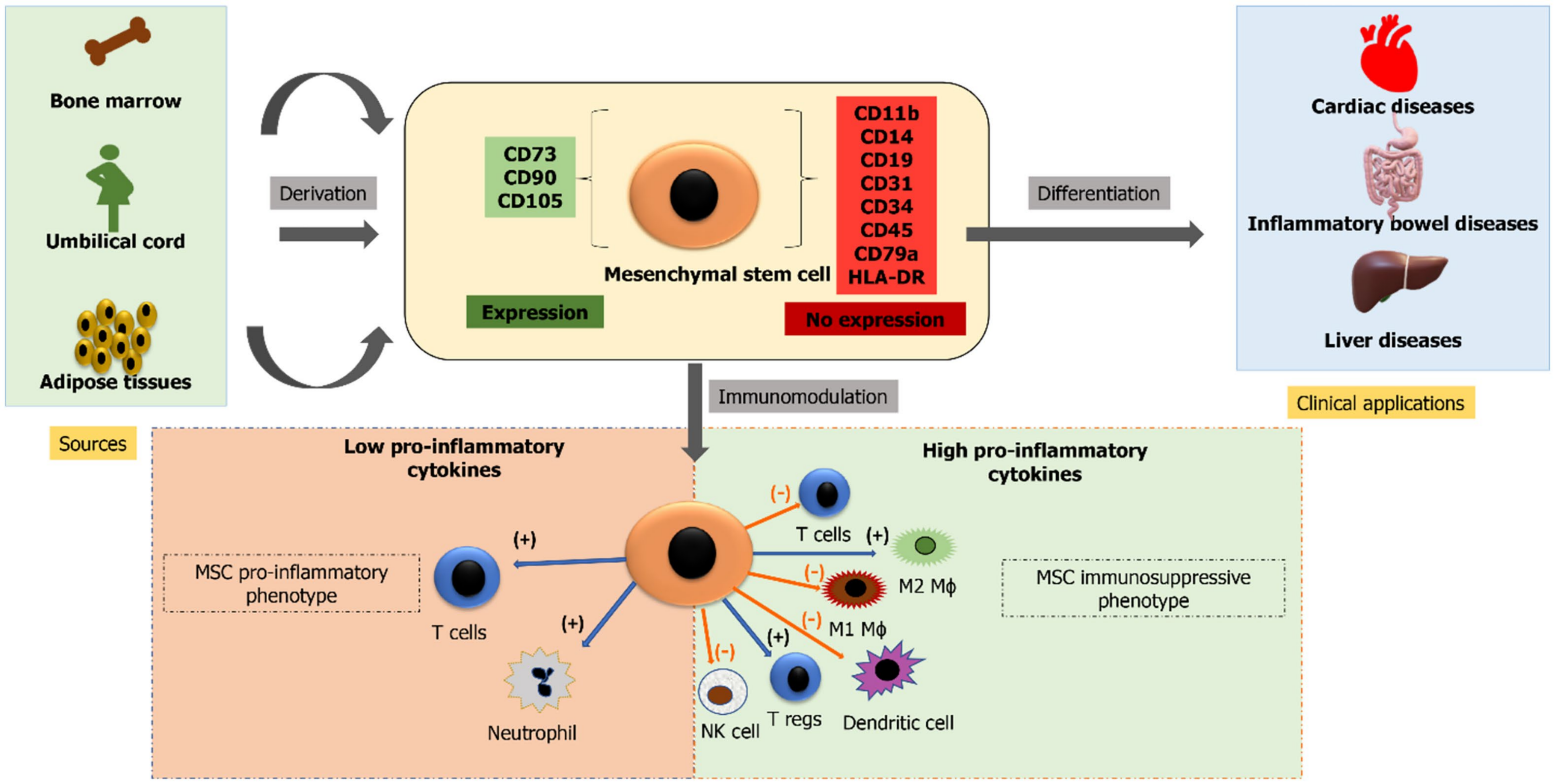
An overview of SCT and clinical application pathway. The figure illustrates the heterogeneity of MSCs derived from the bone marrow, umbilical cord, and adipose tissue, highlighting the differences in marker expression and cytokine-induced shifts in pro-inflammatory and immunosuppressive phenotypes. This further demonstrates how variability in MSC functional properties, including their immunomodulatory capacity and differentiation potential, complicates efforts to achieve consistent and reproducible clinical translation (50). Reprinted from Mukherjee et al. (50), Copyright with permission from Creative Commons Attribution Non Commercial (CC BY-NC 4.0) license.

Figure 2 shows the heterogeneity of umbilical cord, bone marrow, and adipose tissue, which express different characteristic markers, such as, CD73, CD90, and CD105, and lack markers linked to the lineage like CD11b, CD14, CD19, CD34, CD45, and HLA-DR. The figure also illustrates how MSCs can assume pro-inflammatory or immunosuppressive phenotypes, thereby affecting T-cells, macrophages, dendritic cells, NK cells, and neutrophils. The diagram further connects MSC differentiation capacity to clinical use in cardiac, gastrointestinal, and hepatic diseases, highlighting the variability of MSC functionality as a critical constraint for clinical translation (50).

Regular tests that can determine cell viability, differentiation potential, and secretory patterns are also not available, and clinicians lack a real-time measurement tool to gage the strength of products (51). This makes it difficult to interpret clinical studies and can lead to inconsistent findings. Therefore, establishing sensible and attainable quality control procedures for clinical stem cell products is a priority in the field (52).

### 5.2 Regulatory considerations

Regulatory mechanisms developed to regulate cell-based therapies are safety-oriented but create a significant challenge to the innovation and uptake of these therapies. Less manipulated autologous products include fresh bone marrow aspiration or tissue stromal fractions, which typically do not raise as many regulatory issues and can be administered during a single surgical intervention (53). However, Kapoor et al. showed that culture-expanded or genetically modified cell products are considered the same type of regulation as biologics, indicating that they must undergo extensive preclinical and phase clinical studies (54).

These needs increase the cost of development and can limit access to specialized amenities. The varying rules in different countries also make multinational research more challenging and hinder the implementation of artificial intelligence uniformly. Safety, quality assurance, and accessibility can only be achieved through continued cooperation among clinicians, researchers, the industry, and regulatory bodies.

### 5.3 Uncertainty regarding optimal delivery strategies

Though, the schedule, pathway, and dosage of SCT for osteonecrosis remain unclear. The most significant factors are the stage of the disease to be treated, the type of cell to be used with specific etiologies, whether a single treatment is sufficient, and how to combine cells with decompression, grafting, or scaffold-based treatments (55).

Existing studies vary in methodology, complicating the identification of best practices, as they differ in decompression techniques, injection sites, imaging guidance, and follow-up methods (56). Carefully designed comparative and dose-finding trials are required to define the effects of these variables on cell survival, integration and long-term clinical outcomes (57).

### 5.4 Imaging and diagnostic limitations

MRI is necessary for the diagnosis and staging of osteonecrosis, although it does not provide much detail on the biological response to SCT (58). The conventional staging system focuses on lesion size and structural integrity rather than perfusion or cellular activity. Vascularity or cellularity enhancement in the early stages cannot be observed using conventional imaging sequences.

More intricate imaging methods, such as dynamic contrast-enhanced MRI, diffusion-weighted imaging, and quantitative perfusion mapping, capture more early biological responses to regenerative therapy and complementary wireless surveillance technologies are capable of further improving continuous physiological monitoring at home (59, 60). Artamonov et al. suggested that the use of such modalities in clinical trials would help determine imaging biomarkers that predict a positive outcome and would be used to select and time interventions in patients with schizophrenia (7).

### 5.5 Long-term safety

Recent data indicate that mesenchymal stem cell-based therapies for osteonecrosis are safe; however, their long-term safety needs to be determined, especially for engineered or pluripotent cell-based products. The risks include ectopic bone formation, improper tissue differentiation, immune reactions, and, in rare cases, neoplastic transformation. These risks depend on the cell source, extent of manipulation, and local microenvironment of the cells (61).

Long-term follow-up and registry-based surveillance are vital components of responsible clinical care. During an age of technological advancement in the field, as it moves toward more complex engineered constructs and gene-enhanced therapies, closely guarded safety surveillance and open reporting will be required to maintain the confidence of clinicians and patients, as revealed by Jadlowsky et al. (62).

## 6 Future directions

### 6.1 Precision medicine and patient-specific therapy

Precision medicine can enhance SCT by matching personal biological traits with the most appropriate regenerative methods. The number of stem cells, lesion vascularity, and underlying etiologies may vary and affect therapeutic responses; however, existing staging systems do not fully consider these differences (63). Advanced imaging and molecular profiling can identify biomarkers to predict which patients are most likely to respond to marrow- or adipose-derived cell therapies, thereby enabling more selective clinical decisions (64).

As awareness of disease heterogeneity increases, patient stratification may be part of treatment planning. Hereditary and epigenetic factors can help predict the likelihood of successful joint-saving interventions or their failure rates. The application of these tools in clinical care would help shift the treatment process toward personalized management and maximize regenerative effects.

### 6.2 Gene-enhanced MSCs and exosome-based therapies

Another strategy to augment bone regeneration in impaired bone disorders is the use of enhanced MSCs (gene-based). Such modified cells can potentially improve resistance to ischemia and decrease the activity of progenitors in necrotic lesions by enhancing the expression of osteogenic or angiogenic factors (65). As reported by Yang et al., initial results show better bone formation and vascularization with the help of the improved cells compared to the unmodified ones, which provides a reason to develop the cells further (66).

Exosome-based therapies offer a cell-free alternative with enhanced paracrine signaling, eliminating cell survival and engraftment issues associated with cell-based therapies. Exosomes are also associated with molecules that regulate osteogenesis, angiogenesis, and inflammation, and their stability can contribute to the storage and delivery of these molecules (67). Exosomes can serve as a standardized and easily accessible regenerative solution; however, further studies are needed.

### 6.3 Advanced scaffolds and 3D engineered constructs

Sankaranarayanan et al. reported that SCT can enhance the biological and mechanical impact of SCT to the maximum when the scaffolds are in a 3D format to support the necrotic area (68). Engineered biomaterials can reproduce subchondral bone by providing control over porosity, stiffness, and degradation features using modern materials (69). These constructs enhance cell survival and bone deposition when used combined with stem cells and may be beneficial for treating larger or border lesions (70).

The release of exosomes or growth factors can be controlled by the design of scaffolds to promote further regeneration. Regeneration stimulation can be performed through individualized constructs, which increase the conformity between the shape of the lesion and loading profile. Wearable video feedback can also be employed to offer functional rehabilitation (71). These constructs cannot be widely used in clinical practice until further appraisal of their integration, permanency, and immune responses is conducted.

### 6.4 Need for rigorous, standardized clinical trials

Although stem cell treatment for osteonecrosis has shown promising results, several issues hinder further advancements in this area, including divergent research designs, varied cell preparations, and inconsistent reporting of outcomes (72). A large, randomized controlled trial comprising multiple centers, standard classification, uniform cell processing, and verified clinical endpoints is necessary to establish the true efficacy and most effective treatment courses (73). Follow-up studies should be conducted over a prolonged period to determine sustainability and track adverse events in the late stages, as more engineered and genetically modified products are used in clinical settings. Imaging biomarkers and biological assays can also be used to inform trial design and detect early signs of treatment success. Owing to rigorous and standardized evaluations, SCT can be effectively integrated into evidence-based care for osteonecrosis.

## 7 Conclusion

Stem cell therapy is a biologically guided treatment for osteonecrosis that optimally targets deficiencies in osteogenesis, ischemia, and inflammation rather than merely providing mechanical support. The most common, studied, and used MSCs are bone marrow-derived MSCs, which can be replaced by adipose- and blood-derived progenitors in situations of poor marrow quality. New modalities include engineered cell constructs, pluripotent stem cell-derived products and exosomes. Several significant challenges have been raised, including poor-quality cells, regulatory obstacles, confusion over optimal delivery and imaging processes, and a lack of long-term safety data. These challenges must be overcome using an interdisciplinary approach based on basic, translational, and clinical research. As more evidence accumulates, stem cell-based therapies will become essential for joint-saving osteonecrosis treatment.

## References

[1] Lu Y Mai Z Cui L Zhao X. Engineering exosomes and biomaterial-assisted exosomes as therapeutic carriers for bone regeneration. Stem Cell Res Ther. (2023) 14:55. doi: 10.1186/s13287-023-03275-x, 36978165 PMC10053084

[2] Torrecillas-Baena B Casado-Díaz A Gálvez-Moreno MÁ Dorado G Camacho-Cardenosa M Pulido-Escribano V. Clinical potential of mesenchymal stem cell-derived exosomes in bone regeneration. J Clin Med. (2023) 12:4385. doi: 10.3390/jcm12134385, 37445420 PMC10342399

[3] Islam MT Bulut D Sharabidze Z. Regenerative medicine in orthopaedic surgery: pioneering advances and their applications. EMJ Innov. (2024) 9:82–94. doi: 10.33590/emjinnov/FGDS3814

[4] Kaneko K Kaufman M Park-Min KH Stein EM Chen H Sverdlov I. Glucocorticoid-induced osteonecrosis in systemic lupus erythematosus patients. Clin Transl Med. (2021) 11:526. doi: 10.1002/ctm2.526, 34709753 PMC8506634

[5] Moya-Angeler J. Current concepts on osteonecrosis of the femoral head. World J Orthop. (2015) 6:590–601. doi: 10.5312/wjo.v6.i8.590, 26396935 PMC4573503

[6] Burnett JR Vasikaran SD. Cardiovascular disease and osteoporosis: is there a link between lipids and bone? Ann Clin Biochem. (2002) 39:203–10. doi: 10.1258/0004563021902134, 12038594

[7] Artamonov MY Sokov EL. Intraosseous delivery of mesenchymal stem cells for the treatment of bone and hematological diseases. Curr Issues Mol Biol. (2024) 46:12672–93. doi: 10.3390/cimb46110752, 39590346 PMC11592824

[8] Shen F Shi Y. Recent advances in single-cell view of mesenchymal stem cell in osteogenesis. Front Cell Dev Biol. (2022) 9:9. doi: 10.3389/fcell.2021.809918, 35071243 PMC8766509

[9] Weinstein RS Manolagas SC Nicholas RW. Apoptosis of osteocytes in glucocorticoid-induced osteonecrosis of the hip. J Clin Endocrinol Metab. (2000) 85:2907–12. doi: 10.1210/jcem.85.8.6714, 10946902

[10] Konarski W Poboży T Konarska K Śliwczyński A Kotela I Hordowicz M . Osteonecrosis related to steroid and alcohol use—an update on pathogenesis. Healthcare. (2023) 11:1846. doi: 10.3390/healthcare11131846, 37444680 PMC10340773

[11] Yang B Zhu X Peng C Zhou L Wang F Liu Z . Synchronized measurement of electromechanical responses of fabric strain sensors under large deformation. Smart Wearable Technol. (2025) 1:A7. doi: 10.47852/bonviewswt52026022

[12] Åstrand J Aspenberg P. Systemic alendronate prevents resorption of necrotic bone during revascularization. A bone chamber study in rats. BMC Musculoskelet Disord. (2002) 3:19. doi: 10.1186/1471-2474-3-19, 12165099 PMC126216

[13] Cui Q Botchwey EA. Emerging ideas: treatment of Precollapse osteonecrosis using stem cells and growth factors. Clin Orthop Relat Res. (2010) 469:2665–9. doi: 10.1007/s11999-010-1738-1, 21161735 PMC3148382

[14] Wang BL Cheng LM Zhang NF Guo WS Sun W Zhang WJ . Decreased proliferation of mesenchymal stem cells in corticosteroid-induced osteonecrosis of femoral head. Orthopedics. (2008) 31:444. doi: 10.3928/01477447-20080501-33, 19292322

[15] Safarova Y Umbayev B Hortelano G Askarova S. Mesenchymal stem cells modifications for enhanced bone targeting and bone regeneration. Regen Med. (2020) 15:1579–94. doi: 10.2217/rme-2019-0081, 32297546

[16] Jiang F Chen N Zhou Z Shen M Zhou M Zhang W . Human amniotic mesenchymal stromal cells promote bone regeneration via activating endogenous regeneration. Theranostics. (2020) 10:6216–30. doi: 10.7150/thno.45249, 32483449 PMC7255030

[17] Doll JA Crawford SE Reiher FK Pins MR Campbell SC Bouck NP. Thrombospondin-1, vascular endothelial growth factor and fibroblast growth factor-2 are key functional regulators of angiogenesis in the prostate. Prostate. (2001) 49:293–305. doi: 10.1002/pros.10025, 11746276

[18] Lee DE Ayoub N Agrawal DK. Mesenchymal stem cells and cutaneous wound healing: novel methods to increase cell delivery and therapeutic efficacy. Stem Cell Res Ther. (2016) 7:37. doi: 10.1186/s13287-016-0303-6, 26960535 PMC4784457

[19] Kylmaoja E Tuukkanen J Nakamura M. Osteoclasts and remodeling based bone formation. Curr Stem Cell Res Ther. (2016) 11:626–33. doi: 10.2174/1574888X10666151019115724, 26477623

[20] Chen Y Huang Y Li J Jiao T Yang L. Enhancing osteoporosis treatment with engineered mesenchymal stem cell-derived extracellular vesicles: mechanisms and advances. Cell Death Dis. (2024) 15:119. doi: 10.1038/s41419-024-06508-w, 38331884 PMC10853558

[21] Wu T Zhou Y Shi W Guo S Tian H Li W . Translational horizons in stem cell therapy for osteonecrosis of the femoral head: a journey from basic research to clinical practice through bibliometric insights. J Transl Med. (2024) 22:982. doi: 10.1186/s12967-024-05784-6, 39478610 PMC11523765

[22] Kingery MT Strauss EJ Manjunath AK Anil U. Bone marrow mesenchymal stem cell therapy and related bone marrow-derived orthobiologic therapeutics. Curr Rev Musculoskelet Med. (2019) 12:451–9. doi: 10.1007/s12178-019-09583-1, 31749105 PMC6942063

[23] Mylonas KJ Forbes SJ Ferreira-Gonzalez S Schmitt R Hughes J Docherty MH . Cellular senescence inhibits renal regeneration after injury in mice, with senolytic treatment promoting repair. Sci Transl Med. (2021) 13:abb0203. doi: 10.1126/scitranslmed.abb0203, 34011625

[24] Oñate B Ferrer-Lorente R Ballesta-López C Herrero J Ybarra J Díez-Caballero A . The subcutaneous adipose tissue reservoir of functionally active stem cells is reduced in obese patients. FASEB J. (2012) 26:4327–36. doi: 10.1096/fj.12-207217, 22772162

[25] Mazini L Rochette L Amine M Malka G. Regenerative capacity of adipose derived stem cells (ADSCs), comparison with mesenchymal stem cells (MSCs). Int J Mol Sci. (2019) 20:2523. doi: 10.3390/ijms20102523, 31121953 PMC6566837

[26] Gangji V Toungouz M Hauzeur JP. Stem cell therapy for osteonecrosis of the femoral head. Expert Opin Biol Ther. (2005) 5:437–42. doi: 10.1517/14712598.5.4.437, 15934823

[27] Harada Y Tsujimoto S Matsugami H Hisatome I Yoshida A Yamamoto Y. Transplantation of freshly isolated adipose tissue-derived regenerative cells enhances angiogenesis in a murine model of hind limb ischemia. Biomed Res. (2013) 34:23–9. doi: 10.2220/biomedres.34.23, 23428977

[28] Tse HF Lau CP Yiu KH. Bone marrow stem cell therapy for myocardial angiogenesis. Curr Vasc Pharmacol. (2007) 5:103–12. doi: 10.2174/157016107780368299, 17430214

[29] Anderlini P Körbling M. The use of mobilized peripheral blood stem cells from normal donors for allografting. Stem Cells. (1997) 15:9–17. doi: 10.1002/stem.150009, 9007218

[30] Özkurt ZN Acar K Yegˇin ZA Akı ŞZ Yagˇcı M Suyanı E . Factors affecting stem cell mobilization for autologous hematopoietic stem cell transplantation. J Clin Apher. (2010) 25:280–6. doi: 10.1002/jca.20246 20623783

[31] Kim B Yang S Lee J You H Shin H. Fucoidan-induced osteogenic differentiation promotes angiogenesis by inducing vascular endothelial growth factor secretion and accelerates bone repair. J Tissue Eng Regen Med. (2017) 12:e1311–24. doi: 10.1002/term.2509 28714275

[32] Kim W Jang CH Kim G. Bone tissue engineering supported by bioprinted cell constructs with endothelial cell spheroids. Theranostics. (2022) 12:5404–17. doi: 10.7150/thno.74852, 35910797 PMC9330514

[33] Lee K Chan CK Goodman SB Patil N. Cell therapy for bone regeneration—bench to bedside. Journal of biomedical materials research part B: applied. Biomaterials. (2008) 89B:252–63. doi: 10.1002/jbm.b.31199, 18777578

[34] Zigdon-Giladi H Machtei EE Bick T Lewinson D. Co-transplantation of endothelial progenitor cells and mesenchymal stem cells promote neovascularization and bone regeneration. Clin Implant Dent Relat Res. (2013) 17:353–9. doi: 10.1111/cid.12104, 23848193

[35] Müller I Pfister SM Handgretinger R Holzwarth C Vaegler M Greil J . Secretion of angiogenic proteins by human multipotent mesenchymal stromal cells and their clinical potential in the treatment of avascular osteonecrosis. Leukemia. (2008) 22:2054–61. doi: 10.1038/leu.2008.217, 18719618

[36] Kumar P Dhillon MS Shetty VD. Efficacy of orthobiologic adjuvants to core decompression for hip preservation in avascular necrosis hip. J Hip Preserv Surg. (2020) 7:423–38. doi: 10.1093/jhps/hnaa051, 33948198 PMC8081433

[37] Papakostidis C Tosounidis TH Jones E Giannoudis PV. The role of “cell therapy” in osteonecrosis of the femoral head. Acta Orthop. (2015) 87:72–8. doi: 10.3109/17453674.2015.1077418, 26220203 PMC4940596

[38] Wang Z Zhang FQ Wang WJ Sun QM Zhang QL Wang LG. Core decompression combined with autologous bone marrow stem cells versus core decompression alone for patients with osteonecrosis of the femoral head: a meta-analysis. Int J Surg. (2019) 69:23–31. doi: 10.1016/j.ijsu.2019.06.016, 31301432

[39] Wang Y Ma X Chai W Tian J. Multiscale stem cell Technologies for Osteonecrosis of the femoral head. Stem Cells Int. (2019) 2019:1–13. doi: 10.1155/2019/8914569, 30728843 PMC6341242

[40] Fang B Wang J Liu Y Wang C Xie Y Zhang Y . The effects of mechanical stretch on the biological characteristics of human adipose-derived stem cells. J Cell Mol Med. (2019) 23:4244–55. doi: 10.1111/jcmm.14314, 31020802 PMC6533502

[41] Rastogi S Nag HL Shivanand G Rijal L Mohanty S Sankineani SR . Intralesional autologous mesenchymal stem cells in management of osteonecrosis of femur: a preliminary study. Musculoskelet Surg. (2013) 97:223–8. doi: 10.1007/s12306-013-0273-0, 23852661

[42] Brayfield C Rubin JP Marra K. Adipose stem cells for soft tissue regeneration. Handchir Mikrochir Plast Chir. (2010) 42:124–8. doi: 10.1055/s-0030-1248269, 20352575

[43] Bunnell B. A. Adipose Tissue-Derived Mesenchymal Stem Cells. Cells. (2021) 10:3433. doi: 10.3390/cells10123433 34943941 PMC8700397

[44] Yu Y Mo X Yi C Sun B. Stem cell homing-based tissue engineering using bioactive materials. Front Mater Sci. (2017) 11:93–105. doi: 10.1007/s11706-017-0373-0

[45] Li X Li B Liu Y Wang X Zhang R Tan X. Synthesis and evaluation of BMMSC-seeded BMP-6/nHAG/GMS scaffolds for bone regeneration. Int J Med Sci. (2019) 16:1007–17. doi: 10.7150/ijms.31966, 31341414 PMC6643122

[46] Rindone AN Nyberg E Grayson WL. 3D-printing composite polycaprolactone-decellularized bone matrix scaffolds for bone tissue engineering applications. Methods Mol Biol. (2017) 1577:209–26. doi: 10.1007/7651_2017_37 28493213

[47] Qi J Yu T Hu B Wu H Ouyang H. Current biomaterial-based bone tissue engineering and translational medicine. Int J Mol Sci. (2021) 22:10233. doi: 10.3390/ijms221910233, 34638571 PMC8508818

[48] Popa-Wagner A Buga AM Doeppner TR Hermann DM. Stem cell therapies in preclinical models of stroke associated with aging. Front Cell Neurosci. (2014) 8:347. doi: 10.3389/fncel.2014.00347, 25404892 PMC4217499

[49] Zajdel A Kałucka M Kokoszka-Mikołaj E Wilczok A. Osteogenic differentiation of human mesenchymal stem cells from adipose tissue and Wharton’s jelly of the umbilical cord. Acta Biochim Pol. (2017) 64:365–9. doi: 10.18388/abp.2016_1488, 28600911

[50] Mukherjee S Kumar R Yadav G. Recent trends in stem cell-based therapies and applications of artificial intelligence in regenerative medicine. World J Stem Cells. (2021) 13:521–41. doi: 10.4252/wjsc.v13.i6.521, 34249226 PMC8246250

[51] Wu S Wei P Cheng X Lee K Chiou A. Optofluidic platform for real-time monitoring of live cell secretory activities using Fano resonance in gold Nanoslits. Small. (2013) 9:3532–40. doi: 10.1002/smll.201203125, 23606668

[52] Ma CY Liu J Chen H Tse HF Lian Q Zhai Y . Translating mesenchymal stem cell and their exosome research into GMP compliant advanced therapy products: promises, problems and prospects. Med Res Rev. (2023) 44:919–38. doi: 10.1002/med.22002 38095832

[53] Mody H Sutaria DS Miles D. Clinical pharmacology considerations for the “off-the-shelf” allogeneic cell therapies. Clin Pharmacol Ther. (2024) 115:1233–50. doi: 10.1002/cpt.3241, 38501153

[54] Kapoor D Chilkapalli SC Prajapati BG Rodriques P Patel R Singh S . The astonishing accomplishment of biological drug delivery using lipid nanoparticles: an ubiquitous review. Curr Pharm Biotechnol. (2024) 25:1952–68. doi: 10.2174/0113892010268824231122041237, 38265380

[55] Xu Y Jiang Y Xia C Wang Y Zhao Z Li T. Stem cell therapy for osteonecrosis of femoral head: opportunities and challenges. Regenerat Ther. (2020) 15:295–304. doi: 10.1016/j.reth.2020.11.003, 33426232 PMC7770428

[56] Pierce TP Elmallah RK Jauregui JJ Mont MA Nace J Lavernia CJ. A current review of core decompression in the treatment of osteonecrosis of the femoral head. Curr Rev Musculoskelet Med. (2015) 8:228–32. doi: 10.1007/s12178-015-9280-0, 26045085 PMC4596206

[57] Theruvath AJ Lacayo NJ Goodman SB Muehe AM Gassert F Daldrup-Link HE . Tracking cell transplants in femoral osteonecrosis with magnetic resonance imaging: a proof-of-concept study in patients. Clin Cancer Res. (2018) 24:6223–9. doi: 10.1158/1078-0432.ccr-18-1687, 30224340 PMC6295241

[58] Asnis SE Rizzo PF Gould ES Bansal M Bullough PG. Magnetic resonance imaging of the hip after displaced femoral neck fractures. Clin Orthop Relat Res. (1994) 298:191–8. 8118975

[59] Kaur S Acharjee T Das D Bhatia M Sharma S Patel A . Switching on smart care: the ascendancy of wireless technologies in continuous health surveillance. Smart Wearable Technol. (2025) 2025:A13. doi: 10.47852/bonviewSWT52026811

[60] Li SP Padhani AR. Tumor response assessments with diffusion and perfusion MRI. J Magn Reson Imaging. (2012) 35:745–63. doi: 10.1002/jmri.22838, 22434697

[61] Neri S. Genetic stability of mesenchymal stromal cells for regenerative medicine applications: a fundamental biosafety aspect. Int J Mol Sci. (2019) 20:2406. doi: 10.3390/ijms20102406, 31096604 PMC6566307

[62] Jadlowsky JK Hexner EO Marshall A Grupp SA Frey NV Riley JL . Long-term safety of lentiviral or gammaretroviral gene-modified T cell therapies. Nat Med. (2025) 31:1134–44. doi: 10.1038/s41591-024-03478-6, 39833408

[63] Jayawickrama SM Ranaweera PM Pradeep RGGR Jayasinghe YA Senevirathna K Hilmi AJ . Developments and future prospects of personalized medicine in head and neck squamous cell carcinoma diagnoses and treatments. Cancer Rep (Hoboken). (2024) 2024:e2045. doi: 10.1002/cnr2.2045 PMC10961052 38522008

[64] Mallinson DJ Dunbar DR Ridha S Sutton ER Rosa O Dalemans W . Identification of potential plasma microRNA stratification biomarkers for response to allogeneic adipose-derived mesenchymal stem cells in rheumatoid arthritis. Stem Cells Transl Med. (2017) 6:1202–6. doi: 10.1002/sctm.16-0356 28186687 PMC5442839

[65] Zhang J Guan J Qi X Ding H Yuan H Xie Z . Dimethyloxaloylglycine promotes the Angiogenic activity of mesenchymal stem cells derived from iPSCs via activation of the PI3K/Akt pathway for bone regeneration. Int J Biol Sci. (2016) 12:639–52. doi: 10.7150/ijbs.14025, 27194942 PMC4870708

[66] Yang F Cho SW Bogatyrev SR Langer R Mei Y Green JJ . Genetic engineering of human stem cells for enhanced angiogenesis using biodegradable polymeric nanoparticles. Proc Natl Acad Sci USA. (2009) 107:3317–22. doi: 10.1073/pnas.0905432106, 19805054 PMC2840438

[67] Wang X Gong W Li R Wang J Li L. Preparation of genetically or chemically engineered exosomes and their therapeutic effects in bone regeneration and anti-inflammation. Front Bioeng Biotechnol. (2024) 12:9388. doi: 10.3389/fbioe.2024.1329388, 38314353 PMC10834677

[68] Sankaranarayanan A Selvamurugan N Shree Ganesh S Ganesh H Ramanathan B Ramprasad A . Nanogels for bone tissue engineering - from synthesis to application. Nanoscale. (2023) 15:10206–22. doi: 10.1039/d3nr01246h, 37305943

[69] Hutmacher DW Lim TC Schantz JT Tan KC Lam CXF. State of the art and future directions of scaffold-based bone engineering from a biomaterials perspective. J Tissue Eng Regen Med. (2007) 1:245–60. doi: 10.1002/term.24, 18038415

[70] Tharakan S Khondkar S Ilyas A. Bioprinting of stem cells in multimaterial scaffolds and their applications in bone tissue engineering. Sensors. (2021) 21:7477. doi: 10.3390/s21227477, 34833553 PMC8618842

[71] Sookhanaphibarn T. Video-based feedback in table tennis training: toward a wearable learning for undergraduate education. Smart Wearable Technol. (2025) 1:A11. doi: 10.47852/bonviewswt52026365

[72] Grier W Harley B Moy A. Cyclic tensile strain enhances human mesenchymal stem cell Smad 2/3 activation and tenogenic differentiation in anisotropic collagen-glycosaminoglycan scaffolds. Eur. Cells Materials. (2017) 33:227–39. doi: 10.22203/eCM.v033a17, 28319248 PMC5453510

[73] Criner GJ Chen B Hergott CA Casal R Marrujo GX Lamb CR . Improving lung function in severe heterogenous emphysema with the Spiration valve system (EMPROVE). A multicenter, open-label randomized controlled clinical trial. Am J Respir Crit Care Med. (2019) 200:1354–62. doi: 10.1164/rccm.201902-0383OC, 31365298 PMC6884033

[74] Marie PJ Fromigué O. Osteogenic differentiation of human marrow-derived mesenchymal stem cells. Regen Med. (2006) 1:539–48. doi: 10.2217/17460751.1.4.539, 17465848

[75] Statkute L Loh Y Jovanovic B Villa M Spahovic D Oyama Y . Mobilization, harvesting and selection of peripheral blood stem cells in patients with autoimmune diseases undergoing autologous hematopoietic stem cell transplantation. Bone Marrow Transplant. (2007) 39:317–29. doi: 10.1038/sj.bmt.1705579, 17277794

[76] Bastami F Nazeman P Moslemi H Rezai Rad M Sharifi K Khojasteh A. Induced pluripotent stem cells as a new getaway for bone tissue engineering: a systematic review. Cell Prolif. (2016) 50:e12321. doi: 10.1111/cpr.12321 27905670 PMC6529104

[77] Qi X Wang Y Yuan H Xu Z Zhang J Li X . Exosomes secreted by human-induced pluripotent stem cell-derived mesenchymal stem cells repair critical-sized bone defects through enhanced angiogenesis and osteogenesis in osteoporotic rats. Int J Biol Sci. (2016) 12:836–49. doi: 10.7150/ijbs.14809, 27313497 PMC4910602

[78] Wang D Cao H Hua W Gao L Yuan Y Zhou X . Mesenchymal stem cell-derived extracellular vesicles for bone defect repair. Membranes. (2022) 12:716. doi: 10.3390/membranes12070716, 35877919 PMC9315966

[79] Barcena AJR Mishra A Bolinas DKM Martin BM Melancon MP. Integration of electrospun scaffolds and biological polymers for enhancing the delivery and efficacy of mesenchymal stem/stromal cell therapies. Front Biosci (Landmark Ed). (2024) 29:228. doi: 10.31083/j.fbl2906228 38940050 PMC11725061

[80] Kiernan CH Farrell E Brama PAJ Wolvius EB. The immune response to allogeneic differentiated mesenchymal stem cells in the context of bone tissue engineering. Tissue Eng Part B Rev. (2017) 24:75–83. doi: 10.1089/ten.TEB.2017.0175, 28816104

[81] Sauerbier S Schmelzeisen R Stricker A Xavier SP Oshima T Gutwald R . *In vivo* comparison of hard tissue regeneration with human mesenchymal stem cells processed with either the FICOLL method or the BMAC method. Tissue Eng Part C Methods. (2010) 16:215–23. doi: 10.1089/ten.TEC.2009.0269, 19473102

